# Development and validation of prognostic nomogram for lung cancer patients below the age of 45 years

**DOI:** 10.17305/bjbms.2020.5079

**Published:** 2021-06

**Authors:** Lili Dai, Wei Wang, Qi Liu, Tongjia Xia, Qikui Wang, Qingqing Chen, Ning Zhu, Yu Cheng, Ying Yan, Jun Shu, Kaixin Qu

**Affiliations:** 1 Department of Medicine, Funan County People’s Hospital, Anhui, China; 2 Department of Respiratory Medicine, Funan County People’s Hospital, Anhui, China; 3 Department of Endocrinology, Punan Hospital of Pudong District, Shanghai, China; 4 Department of Endocrinology, The First Affiliated Hospital of Anhui Medical University, Anhui, China; 5 Department of Chest Surgery, Anhui Chest Hospital, Anhui, China; 6 Department of Tuberculosis, Anhui Chest Hospital, Anhui, China; 7 Department of Respiratory Medicine, The Second Affiliated Hospital of Xuzhou Medical University, Jiangsu China; 8 Department of Interventional Aulmonary and Endoscopy Center, Anhui Chest Hospital, Anhui, China; 9 Department of Oncology, Anhui Cancer Hospital, Anhui, China; 10 Department of Respiratory Medicine, The Fourth Affiliated Hospital of Anhui Medical University, Anhui, China

**Keywords:** Early-onset lung cancer, prognostic nomogram, overall survival, cancer-specific survival, SEER

## Abstract

This study aimed to establish a nomogram for the prognostic prediction of patients with early-onset lung cancer (EOLC) in both overall survival (OS) and cancer-specific survival (CSS). EOLC patients diagnosed between 2004 and 2015 were retrieved from the Surveillance, Epidemiology, and End Results (SEER) database and further divided into training and validation sets randomly. The prognostic nomobgram for predicting 3-, 5- and 10-years OS and CSS was established based on the relative clinical variables determined by the multivariate Cox analysis results. Furthermore, the predictive performance of nomogram was assessed by concordance index (C-index), calibration curve, receiver operating characteristic (ROC) curve and decision curve analysis (DCA) curve. A total of 1,822 EOLC patients were selected and randomized into a training cohort (1,275, 70%) and a validation cohort (547, 30%). The nomograms were established based on the statistical results of Cox analysis. In training set, the C-indexes for OS and CSS prediction were 0.797 (95% confidence interval [CI]: 0.773-0.818) and 0.794 (95% CI: 0.771-0.816). Significant agreement in the calibration curves was noticed in the nomogram models. The results of ROC and DCA indicated nomograms possessed better predict performance compared with TNM-stage and SEER-stage. And the area under the curve (AUC) of the nomogram for OS and CSS prediction in ROC analysis were 0.766 (95% CI: 0.745-0.787) and 0.782 (95% CI: 0.760-0.804) respectively. The prognostic nomogram provided an accurate prediction of 3-, 5-, and 10-year OS and CSS of EOLC patients which contributed clinicians to optimize individualized treatment plans.

## INTRODUCTION

Cancer is a leading cause of morbidity and mortality worldwide, and among the various types of cancers, lung cancer (LC) has one of the highest incidences of fatality. LC accounts for nearly 27% of cancer deaths in the United States and 20% in the European Union [1]. However, it is encouraging to note that the 5-year survival rate for patients with LC in the United States has increased from 17.2% in 2009 to 21.7% in 2019 [2]. This progress may be attributable to the combination of personalized treatment, screening of high-risk groups, and early diagnosis. It was found that people aged <40 years had a low incidence of LC, which increased yearly to include people aged 75 to 80 years [1]. In clinical trials today, early-onset LC (EOLC) defines LC in patients aged <45 years. These patients comprise approximately 5% of all patients with LC [3]. Unlike elderly patients, genetic cancer factors are considered as the mainstream cause of EOLC [4], which intensifies the need for the accurate prognosis and individualized treatment.

Presently, the tumor-node-metastasis (TNM) staging system, developed by the Union for International Cancer Control and used by the American Joint Commission on Cancer, is widely accepted as the criterion to predict the prognosis of patients with various cancers involving tumor invasion (T), regional lymph nodes (N), and distant metastasis (M) [5]. Since the popularization of TNM staging in the 1970s, major revisions have been made to *TNM Classification of Malignant Tumours. 8th Edition*, which publishes the latest, internationally agreed-on standards to describe and categorize cancer stage [7]. However, the prognostic assessment based on the TNM staging system has limitations and is deficient in predicting prognosis accurately.

The nomogram has been acceptance in the last decade as a unique, reliable method for predicting tumor prognosis [7]. It has been applied in the prognosis prediction of many cancers including gastric cancer, breast cancer, testicular cancer, and so on [8-11]. As a prognostic model, the nomogram assesses significant related risk factors for the prediction. Specifically, the nomogram can produce accurate predictions for overall survival (OS) and cancer-specific survival (CSS) in patients, due to the multiple clinical variables in the calculation. In this study, we utilized nomograms to predict 3-, 5-, and 10-year OS and CSS in patients with EOLC.

## MATERIALS AND METHODS

### Data source and patients

Clinicopathological data and individualized prognostic outcomes in patients with EOLC between 2004 and 2015 were obtained from the Surveillance, Epidemiology, and End Results (SEER) database of the National Cancer Institute using SEER*Stat software (version 8.3.5; SEER 18 Regs Custom Data [with additional treatment fields], November 2018 Sub [1975-2016 varying] database). The identification of EOLC patients was based on the exclusion criteria as follows: (I) patients age >45 years old; (II) patients with multiple primaries tumor; (III) the unknown American Joint Committee on Cancer (AJCC) stage; (IV) the unknown TNM stage; (V) patients without surgery. All the eligible EOLC patients included in this study were randomly assigned into the training and validation sets. Local ethics approval or statements were not required because the clinical data used in this study were obtained from the public-access SEER database and thus, the requirement for informed consent was waived.

### Study variables

**FIGURE S1:**
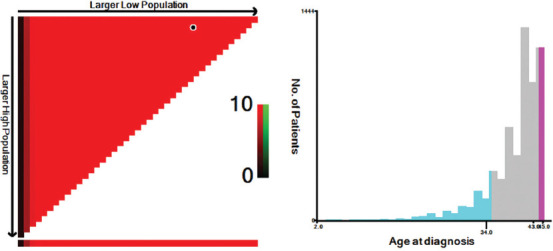
Estimation of the cut-off value for the age determined by X-tile software.

Clinical variables included in this study contained gender, age, race, grade, TNM stage (AJCC, 7th ed.), tumor primary site, SEER stage, chemotherapy and radiotherapy. The age of eligible EOLC patients was divided into three groups (<35, 35-43 and >43; Fig. S1) according to the optimal cut-off value calculated by X-tile software version 3.6.1 (Yale University School of Medicine, US). The tumor primary site contained the following six sites: main bronchus (C34.0), upper lobe (C34.1), middle lobe (C34.2), lower lobe (C34.3), overlapping lesion of lung (C34.8) and not otherwise specified (NOS; C34.9). Moreover, SEER stage comprises three categories: localized, regional, and distant. OS is defined as the time from diagnosis to any cause leading to death or to the date on which data were censored. Moreover, the CSS time analyzed in this study was the survival time from diagnosis to death associated with cancer, excluding other causes. The cut-off point in this study was December 31, 2016.

### Construction and validation of nomogram

Kaplan-Meier curve and log-rank test were performed to investigated the OS and CSS of EOLC patients. Univariate and multivariate regression analyses were used to evaluate the prognostic factors in patients with EOLC. The Cox proportional hazards model was used as the basis for the construction and verification of nomograms. R software version 3.5.1 (http://www.R-project.org) was performed for establishing nomograms. Concordance index (C-index) and calibration curve were performed to evaluate the performance and accuracy of nomograms. The C-index value ranges from 0.50 to 1.00 and shows a positive correlation with the predicted performance of the model. It indicates that and the models accompanied by perfect discrimination ability when the value is 1.00. Moreover, when the calibration curve is applied to a perfectly calibrated model, the prediction will fall on the diagonal 45° in the figure.

In addition, receiver operating characteristic (ROC) curves and decision curve analysis (DCA) were conducted to assess the predicted performance of nanograms, TNM stage, and SEER stage. The statistical software package for social science software (version 20.0; SPSS, Chicago, USA) was applied for all statistical analyses. The results were considered statistically significant as P-value < 0.05 (two-sided).

## RESULTS

### Demographic and pathologic characteristics

**FIGURE 1:**
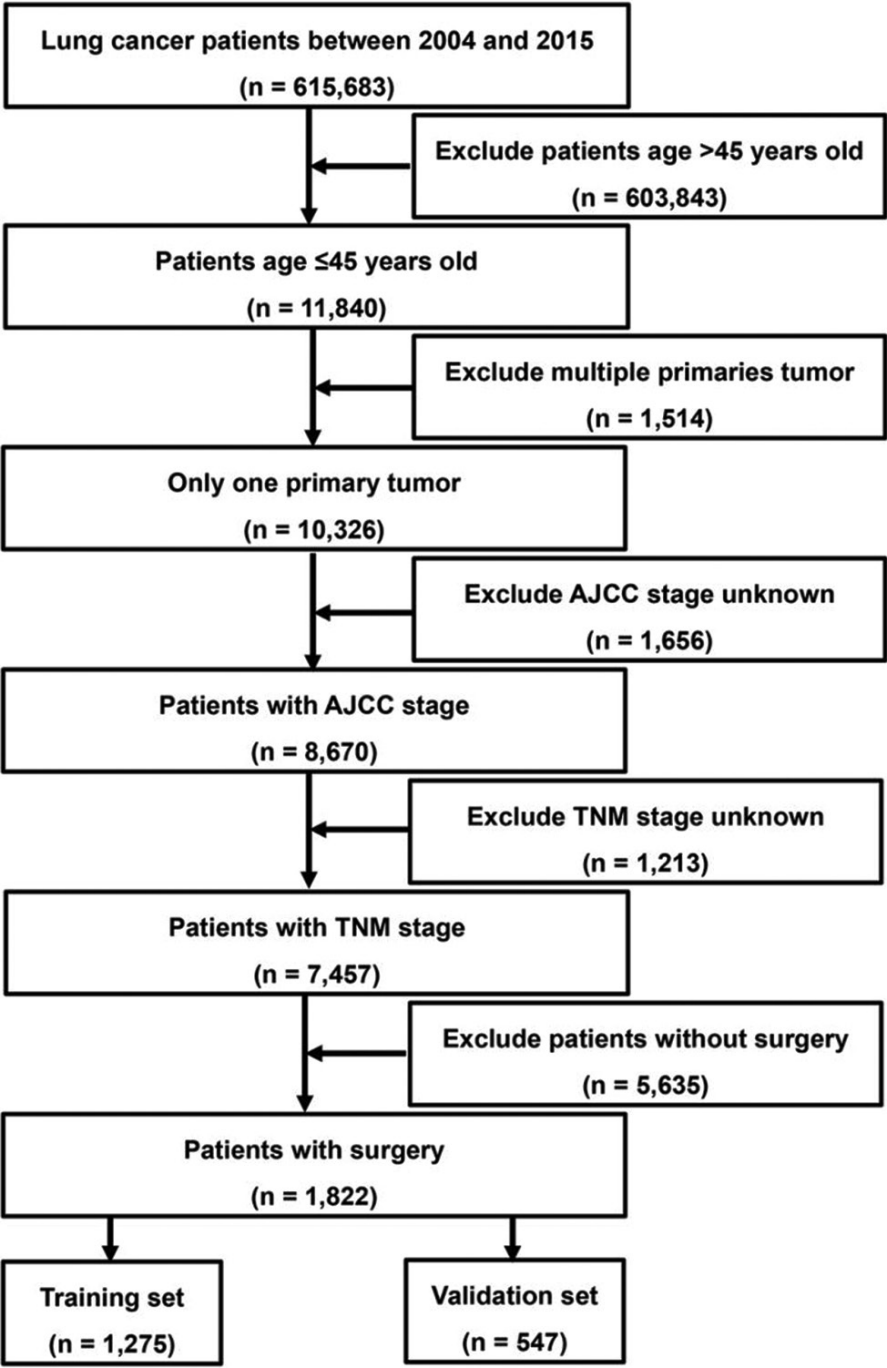
Schematic of specific patient screening process.

The flow process diagram for retrieving patients is shown in Figure 1. Among all 1,822 patients, there were 1068 males (58.6%), 943 patients (51.8%) aged >43 years, and 1381 white patients (75.8%). In addition, the majority of patients in N stage were in N0 stage (1087; 59.7%), whereas 1548 (85.0%) were in M0 stage, according to laboratory examinations and postoperative pathological results. Non-small cell LC (NSCLC) was the most prevalent type of pathology in patients with EOLC, accounting for 67.9% (1237) of patients. The most common primary site of tumor in eligible EOLC patients was the upper lobe (925; 50.8%), followed by the lower lobe (578; 31.7%). The treatment protocol for patients included chemotherapy (874; 48.0%) and radiotherapy (508; 27.9%). The demographic and pathologic characteristics of the patients with EOLC are shown in Table 1.

**TABLE 1:**
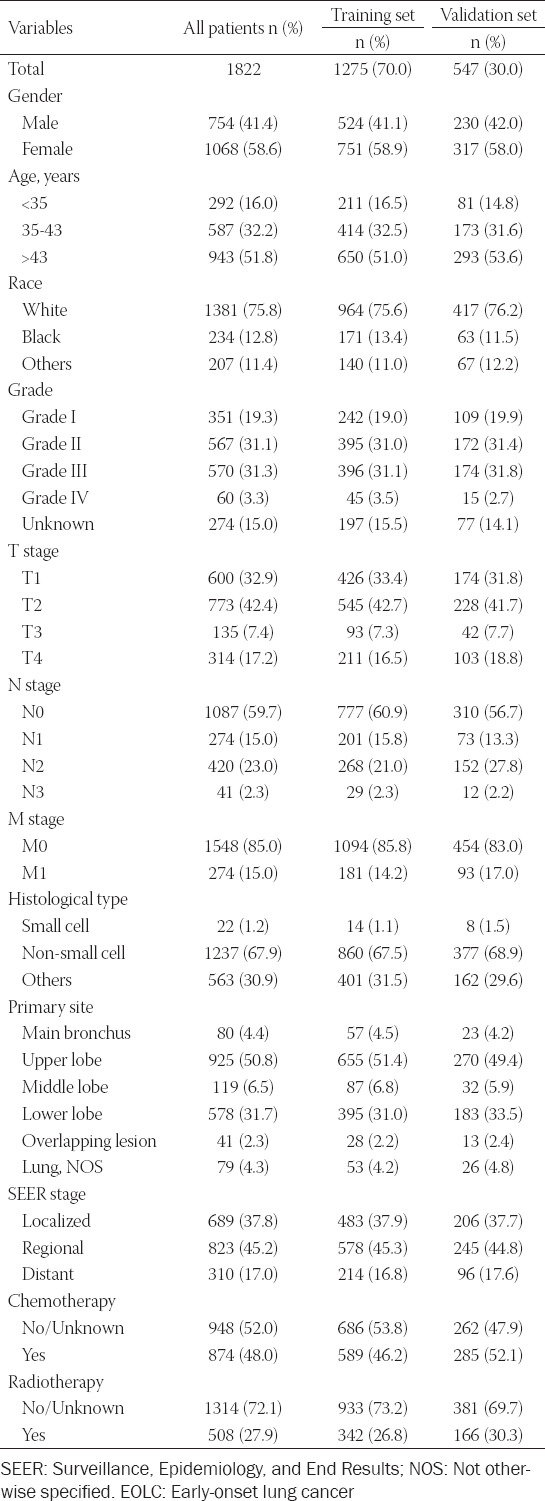
Baseline demographic and clinical characteristics with EOLC patients in our study

### Identification of prognostic factors of OS and CSS

Univariate and multivariate regression analyses were performed to investigate the independent prognostic factors for OS and CSS in patients with EOLC. For OS and CSS, gender, age, race, grade, TNM stage, tumor primary site, SEER stage, chemotherapy, and radiotherapy were the prognostic factors according to the univariate analysis. The multivariate analysis was further applied in our study, and it was found that the three variables (gender, chemotherapy, and radiotherapy) were excluded from the prognostic factors (Table 2). Moreover, the results of multivariate analysis also indicated that age, race, grade, TNM stage, tumor primary site, and SEER stage were independent prognostic factors impacting the CSS in patients with EOLC (Table 3). In addition, we further analyzed prognostic factors in patients with EOLC with NSCLC for their maximum percentage of histological type. The results of multivariate analysis indicated that age, race, grade, TNM stage, and chemotherapy were prognostic factors for OS in patients with EOLC with NSCLC, which lost the chemotherapy for CSS (Table S1).

**TABLE 2:**
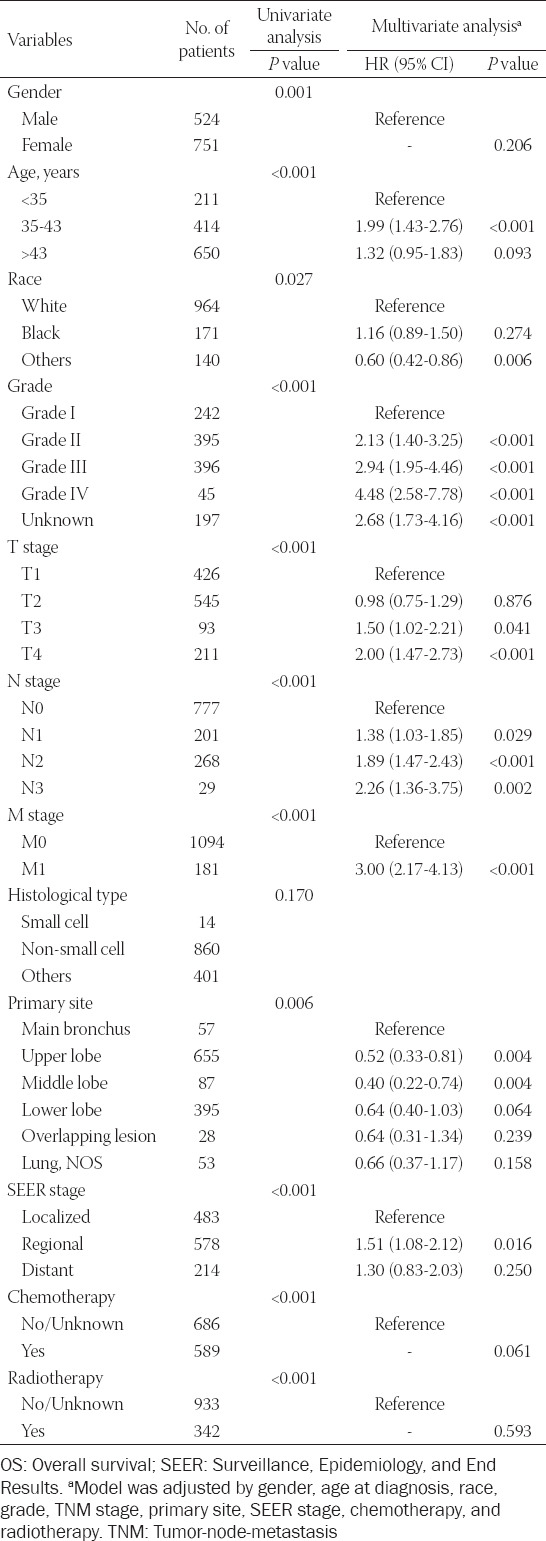
Univariate and multivariate analysis of OS rates

**TABLE 3:**
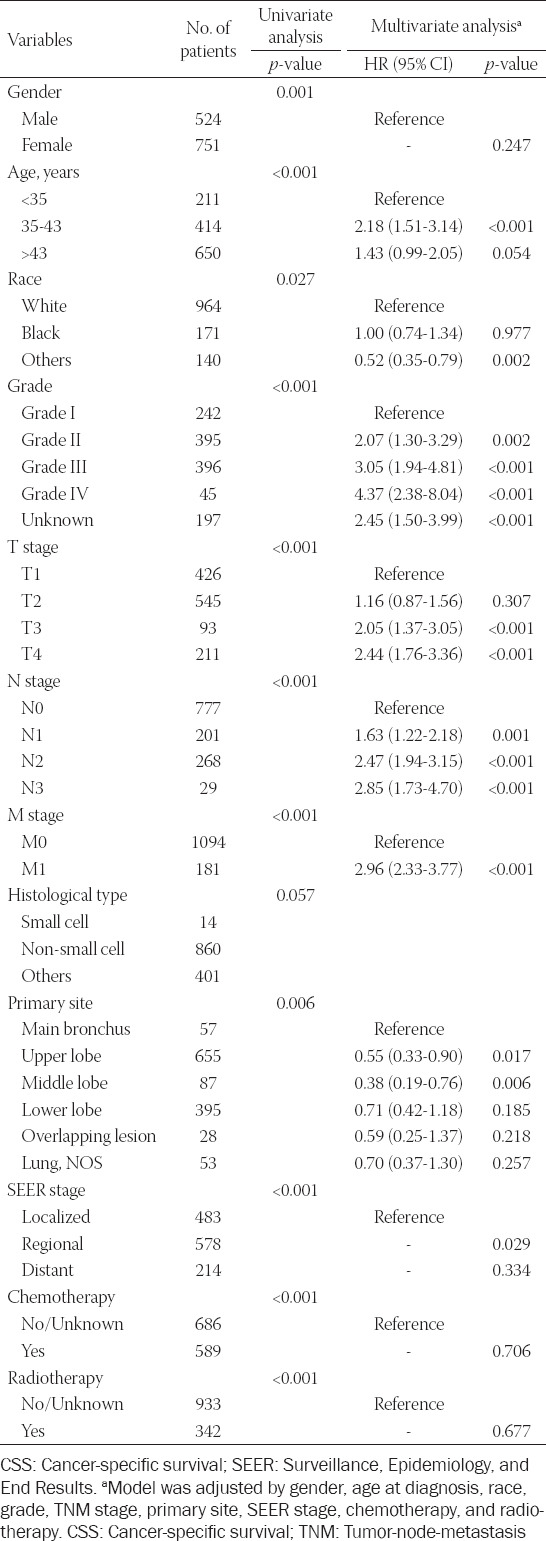
Univariate and multivariate analysis of CSS rates

### Construction and verification of Nomograms

**FIGURE 2:**
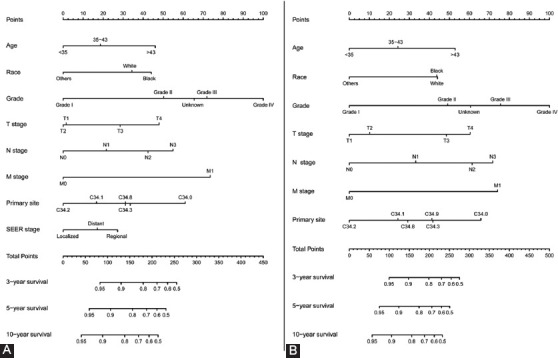
The nomogram containing various factors for the 3-, 5-, and 10-year overall survival (OS) and cancer-specific survival (CSS) prediction of early-onset lung cancer patients. (A) Nomogram for OS; (B) Nomogram for CSS.

**FIGURE S2:**
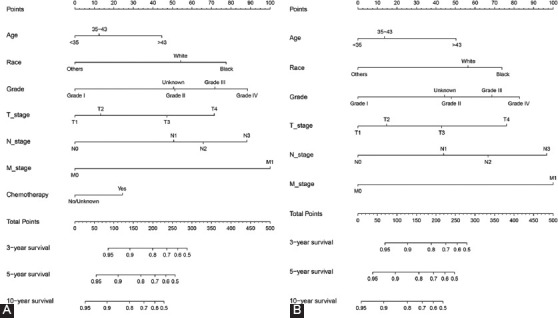
The nomogram containing various factors for the 3-, 5-, and 10-year overall survival (OS) and cancer-specific survival (CSS) prediction of early-onset lung cancer (LC) patients with non-small cell LC. (A) Nomogram for OS; (B) Nomogram for CSS.

The clinical variables included in the construction of nomograms were based on the multivariate Cox regression results. The prognostic nomogram for 3-, 5-, and 10-year OS (Figure 2A) comprised age, race, grade, TNM stage, tumor primary site, and SEER stage as independent prognostic factors, and each variable corresponded to a point according to HR. Moreover, the establishment of a prognostic nomogram for CSS (Figure 2B) included age, race, grade, TNM stage, and tumor primary site as the variables. Simultaneously, the prognostic nomograms for OS (Figure S2A) and CSS (Figure S2B) of patients with EOLC with NSCLC were established according to the Cox regression results.

**FIGURE 3:**
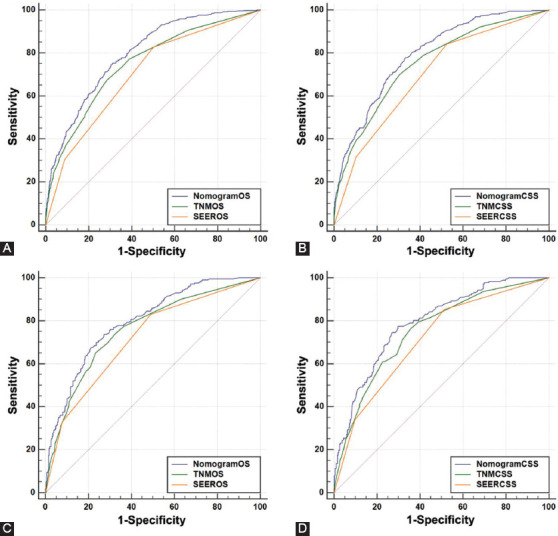
Receiver operating characteristic (ROC) verified the predictive value of nomogram, tumor-node-metastasis stage and surveillance, epidemiology, and end results stage in different sets. (A) ROC for OS in training set; (B) ROC for CSS in training set; (C) ROC for OS in validation set; (D) ROC for CSS in validation set.

**FIGURE 4:**
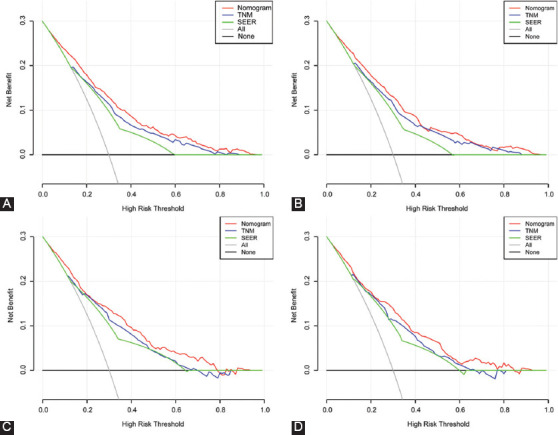
Decision curve analysis (DCA) based on nomograms, tumor-node-metastasis-stage and surveillance, epidemiology, and end results stage in different sets. (A) DCA for OS in training set; (B) DCA for CSS in training set; (C) DCA for OS in validation set; (D) DCA for CSS in validation set.

The time-dependent ROC curves for OS and CSS were plotted to assess the predictive performance of nomograms in different sets. In the training set, the AUC of the nomograms for OS (Figure 3A) and CSS (Figure 3B) was0.766 (95% CI: 0.745–0.787) and 0.782 (95% CI: 0.760–0.804), respectively (Table 4), which were significantly larger than values for TNM stage and SEER stage. The results in validation set showed the same conclusion; the AUC of nomograms were 0.768 (95% CI: 0.738–0.798) for OS (Figure 3C) and 0.780 (95% CI: 0.748–0.812) for CSS (Figure 3D). Simultaneously, the DCA was applied to verify the clinical utility of nomograms. The results indicated that the nomogram showed comparable clinical applicability for predicting OS and CSS as TNM stage and SEER stage, not only in training set (Figure 4A and B) but also in validation set (Figure 4C and D).

**TABLE 4:**
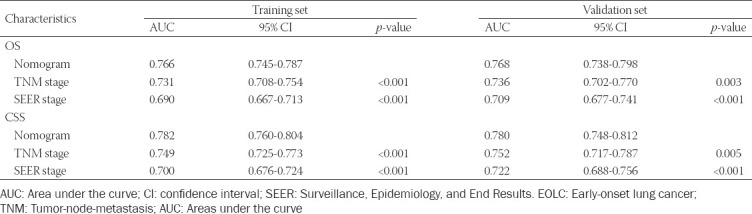
Comparison of AUC between the nomogram, TNM, and SEER stages in EOLC patients

**FIGURE 5:**
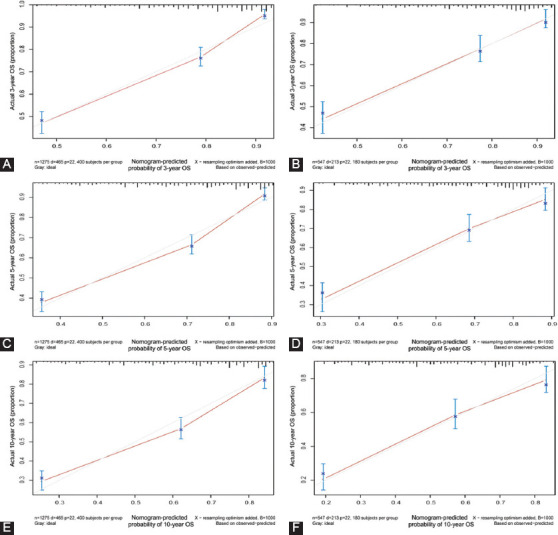
Calibration plot of the 3-, 5-, and 10- year OS nomogram in training and validation sets. (A) 3-year OS in training set; (B) 3-year OS in validation set; (C) 5-year OS in training set; (D) 5-year OS in validation set; (E) 10-year OS in training set; (F) 10-year OS in validation set.

**FIGURE S3:**
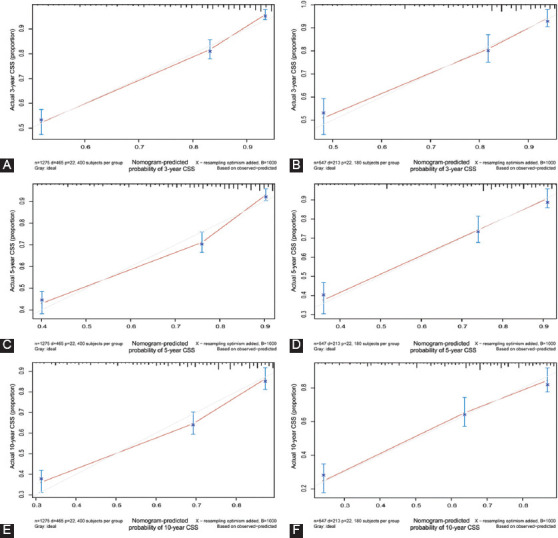
Calibration plot of the 3-, 5-, and 10- year CSS nomogram in training and validation sets. (A) 3-year CSS in training set; (B) 3-year CSS in validation set; (C) 5-year CSS in training set; (D) 5-year CSS in validation set; (E) 10-year CSS in training set; (F) 10-year CSS in validation set.

In addition, the concordance index (C-index) was conducted in this study to verify the nomogram. There were significant differences among nomogram, TNM stage, and SEER stage for OS and CSS (Table 5). We therefore used the calibration curve method to compare nomograms with the perfect curves. The results show that the 3-, 5-, and 10-year OS (Figure 5A, C, and E) and CSS (Figure 5B, D, and F) nomograms in the training set possessed excellent consistency with actual observation, which was also found in the validation set (Figure S3). The above results indicated that there was good agreement between the predictions of the nomograms and the actual observations in both the training set and the validation set.

**TABLE 5:**
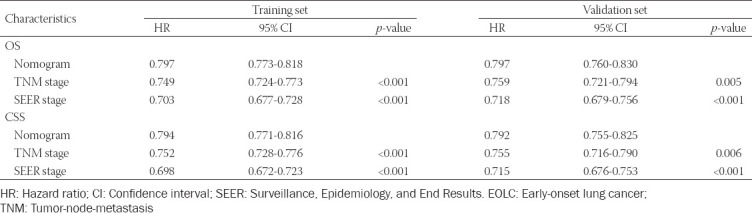
Comparison of C-indexes between the nomogram, TNM, and SEER stages in EOLC patients

## DISCUSSION

At present, the research on patients with EOLC (aged <45 years old) attracted widespread attention due to the rapid increase in LC morbidity and mortality worldwide. It was strongly suggested that genomic mutation was an important predisposing factor for EOLC [12]. Patients with EOLC usually have poor survival outcomes and a higher proportion of family history with other types of cancers [13,14]. In practice, accurately predicting the prognosis of patients with EOLC and formulating individualized treatments are conducive to improving the survival rate. However, the current pathological staging of tumors based on imaging examinations do not meet the requirements for accurate prognosis prediction of patients with EOLC. There is an urgent need for a reliable system to comprehensively consider multiple prognostic factors in patients with EOLC to accurately predict survival time.

This study focused on the prognosis prediction for patients with EOLC based on the construction of nomograms. First, we established prognostic nomograms for 3-, 5-, and 10-year OS and CSS in patients with EOLC. The clinical variables included in the establishment were determined by the results of Cox regression and comprised age, race, grade, TNM stage, tumor primary site, and SEER stage. In addition, the clinical utility and predictive performance of nomograms were verified by ROC curve, DCA curve, and C-index, indicating that efficacy was better than of TNM stage. Furthermore, the accuracy of predicting 3-, 5-, and 10-year OS and CSS was evaluated by the calibration curve, which showed excellent agreement between the nomogram and the actual observation results. In practice, the AUC in ROC analysis and the C-index were generally higher than 0.760 and 0.790, respectively, for all nomograms, which confirmed the promising predictive ability of nomograms. The results of the DCA curve also supported the good clinical practical value of the nomogram.

Nomograms integrated the biological results into a mathematical model to establish a comprehensive consideration of various clinical characteristics and pathological variables of patients with cancer and then graphically displayed the possibility of clinical results. Nomograms were reported to be more accurate than existing models in predicting patient prognosis [15]. Recently, an increased number of nomograms comprising various clinical variables have been used to predict the prognosis of patients with LC [16-19]. Liang et al [18] analyzed NSCLC patient data in multiple clinical centers and established the nomogram for postoperative survival prediction. As a multicenter study, it provided patients with resected NSCLC with an accurate individualized prediction of OS and assisted clinicians in decision making. Similarly, Zheng et al [16] developed the nomogram for predicting prognoses in LC with bone metastasis and comprehensively analyzed the independent prognostic factors, which included age, gender, histological types, grade, and others.

In this study, the following clinical variables, including age, race, grade, TNM stage, tumor primary site, and SEER stage, were the independent risk factors that impacted the prognosis of patients with EOLC. Many studies reported age and race as risk factors for the prognosis of various cancers [20,21]. Genetic differences among races as a significant risk factor for tumor prognosis has also been widely recognized [22,23]. Michele et al [24] found that first-degree relatives of patients with EOLC in black races were more susceptible to developing LC, which indicates significant differences among races.

The grade, primary site, and metastasis of the tumor also significantly affects the prognosis of patients [25]. The pathological grade of a tumor was positively correlated with the degree of malignancy and invasion [26]. It has been suggested that cancer cells in high-grade tumors were insensitive to treatment [27], which adversely affects the prognosis of patients. Tumor site of cancers is as important a factor affecting the prognosis of patients [28,29]. For patients with LC, the primary site of tumor in the right and left lower lobe or in the right middle and left lingual lobe is more susceptible to mediastinal lymph node tumor metastasis [30]. Moreover, lymph node metastasis or distant tumor metastasis represents a poor prognosis and short survival time for patients. In our study, the same results were supported by statistical analysis.

Currently, TNM stage, determined by laboratory results and postoperative pathological examination, is the most widely accepted tumor staging system. In practice, clinicians would judge TNM stage based on individual characteristics of the tumor (T), node (N), and metastasis (M) [6]. Chen et al [31] verified the prognostic value of the *TNM Classification of Malignant Tumours. 8th Edition* TNM staging system for patients with LC and found recurrence-free survival could also be predicted through TNM stage. However, the TNM stage has limitations and could not provide clinicians with individualized prognosis prediction. As shown in this study, patient prognosis was also closely related to a variety of clinical variables except TNM staging, and accurate prediction relied on the comprehensive consideration of all independent risk factors. We successfully established an effective nomogram based on age, race, grade, TNM stage, tumor primary site, and SEER stage, which has been proven a better predictive tool than TNM stage alone. The construction of nomograms would be useful in helping to develop personalized treatment for patients with EOLC.

There are still some limitations to our study. First, the SEER database as a retrospective database includes biases in data collection due to manual recording and other reasons. Second, the clinical data were incomplete; for example, the SEER database failed to record the genetic changes in the patients. Third, the analyzed data did not represent other regions and required external verification. Therefore, it is necessary to conduct multicenter prospective clinical trials to verify the accuracy of nomograms.

## CONCLUSIONS

We established prognostic nomograms for 3-, 5-, and 10-year OS and CSS in EOLC patients based on a large amount of clinical data, and this prognostic nomogram has good predictive ability. The models could help clinicians prepare personalized treatment for patients with EOLC.

## Figures and Tables

**TABLE S1:**
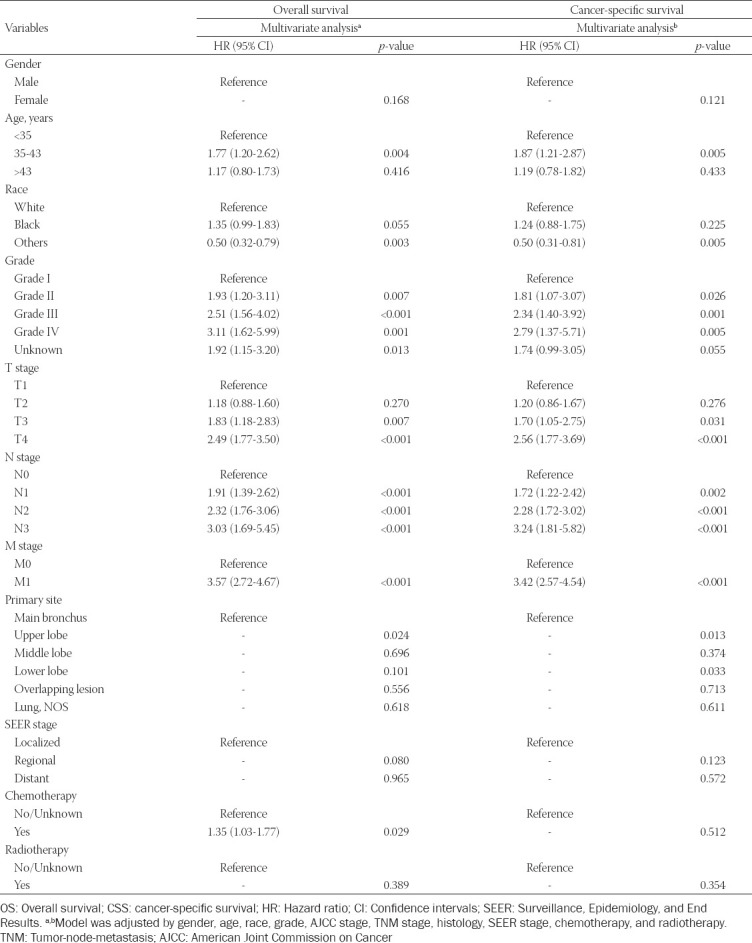
Multivariate analysis of OS and CSS rates of patients with non-small cell lung cancer in the training set
